# Expression and prognostic significance of the polymeric immunoglobulin receptor in esophageal and gastric adenocarcinoma

**DOI:** 10.1186/1479-5876-12-83

**Published:** 2014-04-02

**Authors:** Richard Fristedt, Alexander Gaber, Charlotta Hedner, Björn Nodin, Mathias Uhlén, Jakob Eberhard, Karin Jirström

**Affiliations:** 1 Department of Clinical Sciences, Oncology and Pathology, Lund University, Skåne University Hospital, 221 85 Lund, Sweden; 2 Science for Life Laboratory, Royal Institute of Technology, 171 21 Stockholm, Sweden; 3 School of Biotechnology, AlbaNova University Center, Royal Institute of Technology, 106 91 Stockholm, Sweden

**Keywords:** Polymeric immunoglobulin receptor, Esophageal adenocarcinoma, Gastric adenocarcinoma, Gastroesophageal junction adenocarcinoma, Barrett’s esophagus, Intestinal metaplasia, Prognosis

## Abstract

**Introduction:**

The polymeric immunoglobulin receptor (PIGR) has been proposed to be a candidate prognostic biomarker in a few cancer forms, and one previous study reported that reduced PIGR expression signifies more aggressive tumours of the distal esophagus and gastroesophageal junction (GEJ). In the present study, we examined the expression, clinicopathological correlates and prognostic significance of PIGR expression in an extended cohort of adenocarcinoma of the upper gastrointestinal tract.

**Materials and methods:**

Immunohistochemical PIGR expression was examined in a consecutive cohort of patients with surgically resected, radio-chemonaive adenocarcinoma of the esophagus, GE-junction and stomach (n = 173), including paired samples of benign-appearing squamous epithelium (n = 51), gastric mucosa (n = 114), Barrett’s esophagus (BE) or intestinal metaplasia (IM) (n = 57) and lymph node metastases (n = 75). Non-parametric tests were applied to explore associations between PIGR expression in primary tumours and clinicopathological characteristics. Classification and regression tree analysis was applied for selection of prognostic cut-off. The impact of PIGR expression on overall survival (OS) and recurrence-free survival (RFS) was assessed by Kaplan-Meier analysis and hazard ratios (HR) calculated by adjusted and unadjusted Cox proportional hazards modelling.

**Results:**

PIGR expression was significantly higher in intestinal metaplasia (BE or gastric IM) compared to normal tissues and cancer (p < 0.001). Reduced PIGR expression in primary tumours was significantly associated with more advanced tumour stage (p = 0.002) and inversely associated with involved margins (p = 0.034). PIGR expression did not differ between primary tumours and lymph node metastases. There was no significant difference in PIGR expression between tumours with and without a background of intestinal metaplasia. High PIGR expression was an independent predictor of a prolonged OS (HR = 0.60, 95% CI 0.36-0.99) and RFS (HR = 0.49, 95% CI 0.27-0.90) in patients with radically resected (R0) primary tumours and of an improved RFS (HR = 0.32, 95% CI 0.15-0.69) in curatively treated patients with R0 resection/distant metastasis-free disease.

**Conclusion:**

High PIGR expression independently predicts a decreased risk of recurrence and an improved survival in patients with adenocarcinoma of the upper gastrointestinal tract. These findings are of potential clinical relevance and merit further validation.

## Introduction

The incidence and death rates from gastric cancer are steadily decreasing in the westernized world, but it still remains the second most common cause of cancer death worldwide [1]. In contrast, there has been a 2.5-fold increase of gastro-esophageal junction (GEJ) adenocarcinoma (AC) over the last four decades [2]. The increase is attributable at least in part to the known risk factors for development of GEJAC; smoking, obesity and GE reflux disease. Esophageal carcinoma rates are also increasing and it is now the eighth most common cancer worldwide [3-5]. As for GEJAC, there is a sharp increase for esophageal adenocarcinoma and the incidence now surpasses squamous cell carcinoma in Europe and America [4,6]. The late onset of symptoms, e.g. dysphagia, and the early spread to regional lymph nodes explain the still dismal 5-year survival rates of 15-25% [3,7] and there is an apparent need for improved prognostic and treatment predictive markers in upper gastrointestinal tract carcinomas as a group.

The polymeric immunoglobulin receptor (PIGR) is a member of the immunoglobulin superfamily and transports immunoglobulin A (IgA) onto mucosal surfaces. PIGR binds polymeric IgA at the basolateral surface of epithelial cells and the complex is then transcytosed to the apical cell surface, where the extracellular part of PIGR is cleaved off as a secretory component (SC) bound to polymeric IgA. The extracellular component of PIGR can also be cleaved off to produce SC without being bound to IgA molecules and then acts as a scavenger on the mucosal lining [8].

PIGR has been described as a putative cancer biomarker in a few studies on different cancer forms, the majority of which indicate an association between low PIGR expression and more aggressive disease. In a small case series (n = 42) Gologan et al. found PIGR-negative adenocarcinomas in the distal esophagus and GEJ to be associated with lymph node metastasis and a trend towards reduced survival [9]. Low PIGR expression has also been shown to correlate with progression from colon adenoma to carcinoma [10] and with poor prognosis in colon cancer [11]. Furthermore, loss of PIGR expression has been linked to tumour progression in non-small cell lung cancer [12] while overexpression of PIGR has been associated with the less aggressive type 1 endometrial cancer [13] as well as correlating with a better prognosis in bladder cancer [14] and epithelial ovarian cancer [15]. However, contradicting data was reported in a study on hepatitis B-derived hepatocellular carcinoma, where high PIGR expression was found to be associated with greater metastatic potential and poor prognosis [16].

The aim of this study was to examine the expression and prognostic impact of PIGR in a consecutive cohort of adenocarcinoma of the esophagus, GEJ and stomach (n = 173).

## Methods

### Study design and participants

The study comprised a consecutive cohort of 303 patients with esophageal and gastric adenocarcinomas who had been surgically treated in the university hospitals of Lund and Malmö from Jan 1st 2006 – Dec 31st 2010. A total number of 128 patients were excluded; all patients who had received neoadjuvant treatment (n = 31), cases with metastases from other cancers (n = 12), mucosal resections (n = 6), consultancies from other departments (n = 22), cases with missing archival specimens (n = 2) and double/incorrectly coded cases (n = 55). The selected tumours were histopathologically re-examined, including confirmation of diagnosis and number of lymph nodes with metastasis (re-classified following the standardized TNM 7 classification). Clinical data, and information on recurrence, vital status and cause of death was obtained from the medical charts.

Patient and tumour characteristics are described in Table 1.

**Table 1:** Patient and tumour characteristics in the entire cohort and according to tumour location

**Factor**	**Entire cohort (n = 175) n (%)**	**Esophagus (n = 60) n (%)**	**GE-junction (n = 45) n (%)**	**Stomach (n = 66) n (%)**	P*
**Age**					
Mean	70.2	76.9	69.9	72.0	0.080
Median	69.8	66.02	68.7	72.6	
(Range)	42.6-94.4	48.2-88.5	48.7-88.5	42.6-94.4	
**Sex**					
Women	41 (23.4)	6 (10.0)	12 (26.7)	20 (30.3)	0.007
Men	134 (76.6)	54 (90.0)	33 (73.3)	46 (69.7)	
**T stage**					
1	19 (11.0)	9 (15.3)	3 (6.8)	6 (9.2)	0.265
2	32 (18.6)	10 (16.9)	4 (9.1)	17 (26.2)	
3	94 (54.7)	34 (57.6)	33 (75.0)	26 (40.0)	
4	27 (15.7)	6 (10.2)	4 (9.1)	16 (24.6)	
Missing	3	1	1	1	
**Resection margins**					
R0	122 (69.7)	38 (63.3)	30 (66.7)	51 (77.3)	0.016
R1	34 (19.4)	10 (16.7)	11 (24.4)	12 (18.2)	
R2	19 (10.9)	12 (20.0)	4 (8.9)	3 (4.5)	
**Examined nodes**					
Mean	29.0	36.6	29.7	25.8	<0.001
Median	30.2	33.5	28.00	23.0	
Range	1-112	10-72	8-48	1-112	
Missing	14	2	1	11	
**N stage**					
0	59 (33.7)	15 (25.0)	12 (26.7)	28 (42.4)	0.032
1	30 (17.1)	11 (18.3)	7 (15.6)	12 (18.2)	
2	41 (23.4)	15 (25.0)	14 (31.1)	12 (18.2)	
3	45 (25.7)	19 (31.7)	12 (26.7)	14 (21.2)	
**M stage**					
0	137 (88.4)	51 (86.4)	40 (88.9)	45 (91.8)	0.377
1	18 (11.6)	8 (13.6)	5 (11.1)	4 (8.2)	
Missing	20	1		19	
**Differentiation grade**					
High	6 (4.0)	3 (5.9)	1 (2.5)	1 (1.8)	0.002
Intermediate	40 (26.8)	21 (41.2)	9 (22.5)	9 (16.4)	
Low	103 (69.1)	27 (52.9)	30 (75.0)	45 (81.8)	
Missing	26	9	5	11	
**Adjuvant radio/chemotherapy**					
No	150 (85.7))	54 (93.1)	39 (90.7)	55 (85.9)	0.196
RT	1 (0.6)	1	0	0	
CT with oxaliplatin	2 (1.1)	0	0	2 (3.1)	
CT without oxaliplatin	3 (1.7)	0	2 (4.7)	1 (1.6)	
RT + CT without oxaliplatin	6 (3.4)	2 (3.4)	2 (4.7)	2 (3.1)	
RT + CT, NOS	2	0	0	2 (3.1)	
Yes, NOS	3 (1.7)	1 (1.7)	0	2 (3.1)	
Unknown	8	2	2	2	
**Location**					
Esophagus	60 (35.1)	-	-	-	
GE-junction	45 (26.3)	-	-	-	
Stomach	66 (38.6)	-	-	-	
Unknown	4				
**Follow-up**					
Mean	2.92	2.97	2.87	2.92	0.848
Median	2.27	2.65	2.17	2.15	
Range	0.01-7.70	0.26-7.70	0.01-7.64	0.03-7.60	
**Vital status**					
Alive	64 (36.6)	27 (45.0)	14 (31.1)	22 (33.3)	0.184
Dead	111 (63.4)	33 (55.0)	31 (68.9)	44 (66.7)	
**Recurrence**					
No	64 (46.4)	24 (46.2)	14 (38.9)	25 (50.0)	0.705
Yes	74 (53.6)	28 (53.8)	22 (61.1)	25 (50.0)	
Unknown	37	8	9	16	

*Chi-square test and Fisher’s Exact test was applied for analysis of differences in the distribution of clincipathological characteristics according to tumour location, not including the entire cohort.

Ethical permission was received from the regional ethical board of Lund University (ref nr 445/07).

### Tissue microarray construction

Tissue microarrays (TMAs) were constructed using a semi-automated arraying device (TMArrayer, Pathology Devices, Westminister, MD, USA). Duplicate tissue cores (1 mm) were obtained from primary tumours. In addition, lymph node metastases were sampled in 81 cases, intestinal metaplasia (IM), either Barrett’s esophagus (BE) or gastric IM, in 73 cases, normal squamous epithelium in 96 cases and normal gastric mucosa in 131 cases. Duplicate cores were obtained from different blocks of the primary tumour and different lymph node metastases in cases with more than one metastasis. Normal squamous epithelium and gastric mucosa was represented in single cores, and intestinal metaplasia in 1–3 cores.

### Immunohistochemistry and staining evaluation

For immunohistochemical analysis of PIGR expression, 4 μm TMA-sections were automatically pre-treated using the PT Link system and then stained in an Autostainer Plus (DAKO; Glostrup, Copenhagen, Denmark) with a polyclonal, monospecific antibody; HPA012012, Atlas Antibodies AB, diluted 1:200. The specificity of the antibody was confirmed by immunofluorescence, Western blotting and protein arrays (http://www.proteinatlas.org).

PIGR was exclusively expressed in the cytoplasm and cell membrane. The staining was annotated by two observers (RF, AG) whereby consensus for each core was reached in estimated fraction 0.0-1.0 (1 = 100%) of stained cells, while staining intensity was annotated as 0 = negative, 1 = weak, 2 = moderate and 3 = strong intensity. A multiplier of intensity (0–3) and fraction (0.0-1.0) for each core was calculated and a mean value of all annotated cores was used in the analyses.

### Statistical analysis

Non-parametric Mann–Whitney U and Kruskal-Wallis tests were applied for analyses of differences in the distribution of PIGR expression according to clinicopathological characteristics, in the entire cohort and according to tumour location. The Chi-square test and Fisher’s Exact test were used to analyse differences in the distribution of clinicopathological characteristics according to tumour location. Classification and regression tree (CRT) analysis [17] was used to assess optimal prognostic cut off for PIGR expression in overall survival (OS) and recurrence free survival (RFS). Kaplan Meier analysis and the log rank test were applied to estimate differences in OS and RFS in strata according to high and low PIGR expression. RFS was defined from the date of surgery to the date of locoregional or distant recurrence. Cox regression proportional hazard’s modelling was used to estimate the impact of PIGR expression on OS and RFS in both unadjusted analysis and in a multivariable model adjusted for, age, sex, T-stage, N-stage, M-stage, differentiation, resection margins and tumour location. Some subjects had no information on one or several markers and missing values were coded as a separate category for categorical variables. Missing values for categorical variables co-varied and the adjusted model did not converge due to many constant values. In order to avoid this, only patients with information on PIGR expression were included in the adjusted analysis. A backward conditional method was used for variable selection in the adjusted model. All test were two sided. P-values <0.05 were considered significant. All statistical analyses were performed using IBM SPSS Statistics version 22.0 (SPSS Inc., Chicago, IL, USA).

## Results

### PIGR expression in normal tissues, intestinal metaplasia, primary tumours and lymph node metastases

**Figure 1:**
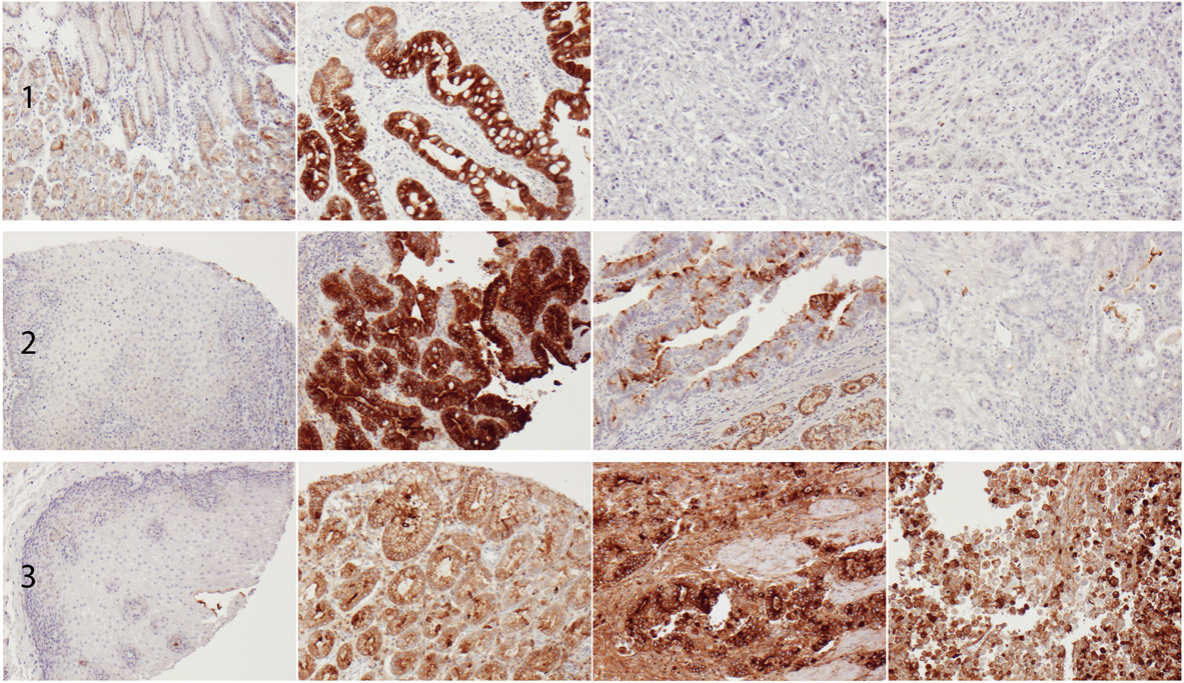
**Sample immunohistochemical images of PIGR staining.** Images (10× magnification) of PIGR expression in different tissue entities from three cases. The mean score corresponds to the value of the sum of the fraction × intensity of all annotated cores. From left to right: **(1)** normal gastric mucosa (mean/total score 0.70), intestinal metaplasia (mean/total score 3), primary tumour (mean/total score 0.2) and metastasis (mean/total score 0) in a T3N3M1 gastric cancer, **(2)** squamous epithelium (mean/total score 0), Barrett’s esophagus (mean/total score 3), two cores from primary tumour (score 2 and 0.2, respectively, mean/total score 1.1) in a T2N2M0 esophageal cancer, **(3)** squamous epithelium (mean/total score 0), normal gastric mucosa (mean/total score 2.0), and two cores from primary tumour (both score 3, mean/total score 3) from a T3N3M0 GE-junction cancer.

**Figure 2:**
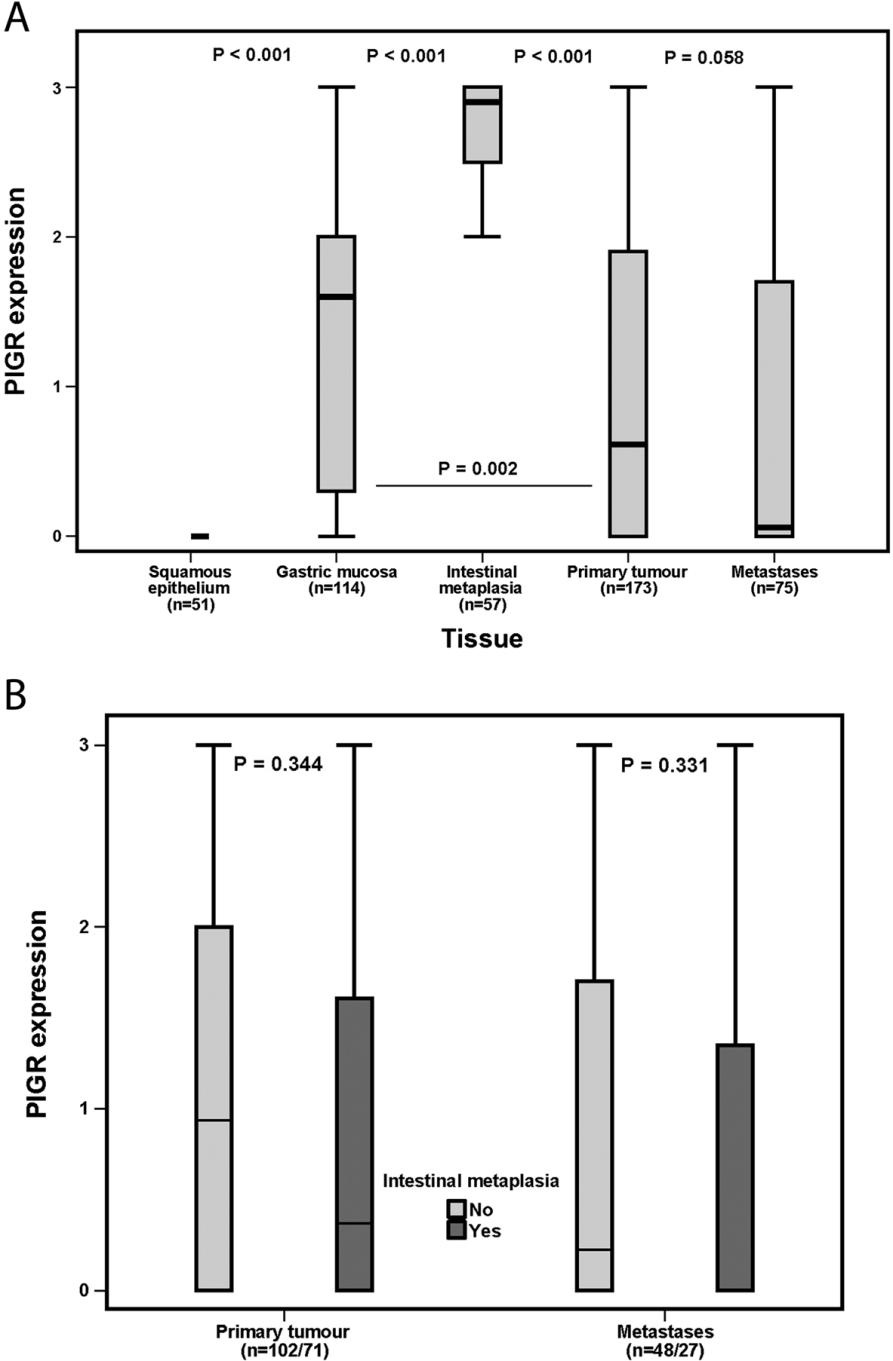
**PIGR expression in normal tissues, intestinal metaplasia, primary tumours and metastases. (A)** Box plots visualizing the distribution of PIGR expression (total score) in normal squamous epithelium, intestinal metaplasia (Barrett’s esophagus or gastric intestinal metaplasia), primary tumours and metastases in the entire cohort, and **(B)** primary tumours and metastases in cases with and without reported Barrett’s esophagus/intestinal metaplasia, respectively.

Sample IHC images are shown in Figure 1 and the distribution of PIGR staining (total score of fraction × intensity) was evaluated in normal tissues, IM, primary tumours and lymph node metastases is shown in Figure 2. All samples of squamous epithelium (=51) were negative for PIGR expression, in contrast to IM (n = 57) where PIGR expression was significantly higher than in all other tissues. In mucosa with IM, PIGR was strongly expressed in the majority of the cells, not only goblet cells, irrespective of the anatomical origin, i.e. BE or gastric IM, and of the presence or absence of dysplasia. In normal gastric mucosa (n = 114), PIGR was expressed both in the glandular cells and in the columnar epithelium in various fractions but with all over weaker intensity than in IM. PIGR expression could be evaluated in 173/175 (98,9%) of the primary tumours and in 75/81 (92.6%) of the sampled lymph node metastases. A total number of 47/173 (27.2%) of primary tumours and 32/75 (42.7%) of lymph node metastases were negative for PIGR expression. There was no heterogeneity between duplicate tissue cores in negative and strongly positive cases. PIGR expression did not differ significantly between primary tumours and lymph node metastases, although a tendency towards lower expression was seen in lymph node metastases (p = 0.058, Figure 2A). As shown in Figure 2B there was no significant difference in PIGR expression between primary tumours or lymph node metastases in cases with or without associated IM.

### Associations of PIGR expression with clinicopathological characteristics

As demonstrated in Table 2 there was a significant association between reduced PIGR expression and a more advanced T-stage (p = 0.002) and involved resection margins (p = 0.034) in the entire cohort. There were no significant associations between PIGR expression and any other clinicopathological parameters in the entire cohort. The significant association of PIGR with T-stage was retained in esophageal cancer (p = 0.006), while in gastric cancer, PIGR expression was significantly associated with a more advanced N-stage (p = 0.043).

**Table 2:** Associations of PIGR expression in primary tumours with clinicopathological parameters in the entire cohort and according to tumour location

**Factor**	**Entire cohort median (range)**	***p-value***	**Esophagus median (range)**	***p-value***	**GE-junction median (range)**	***p-value***	**Stomach median (range)**	***p-value***
**Age**								
≤ average	0.610(0.00-3.00)	0.347	1.059(0.00-3.00)	0.217	0.010(0.00-2.75)	0.034	1.250(0.00-3.00)	0.538
>average	0.605(0.00-3.00)		0.120(0.00-3.00)		1.200(0.00-3.00)		0.987(0.00-3.00)	
**Gender**								
Female	0.375(0.00-3.00)	0.817	0.275(0.00-2.85)	0.816	0.330(0.00-3.00)	0.570	0.762(0.00-3.00)	0.915
Male	0.810(0.00-3.00)		0.375(0.00-3.00)		0.342(0.00-3.00)		1.225(0.00-3.00)	
**T-stage**								
T1	1.930(0.00-3.00)	0.002	2.031(0.12-2.70)	0.006	1.610(1.60-1.96)	0.154	1.970(0.80-3.00)	0.157
T2	1.100(0.00-3.00)		1.150(0.00-3.00)		0.225(0.00-2.20)		1.250(0.00-3.00)	
T3	0.200(0.00-3.00)		0.062(0.00-2.85)		0.342(0.00-3.00)		0.370(0.00-3.00)	
T4	0.128(0.00-3.00)		0.050(0.00-2.18)		0.000(0.00-1.06)		0.717(0.00-3.00)	
**N-stage**								
N0	1.200(0.00-3.00)	0.193	1.560(0.00-2.70)	0.065	1.361(0.00-3.00)	0.221	1.150(0.00-3.00)	0.043
N1	0.120(0.00-3.00)		0.040(0.00-3.00)		1.610(0.00-2.75)		0.023(0.00-2.03)	
N2	0.375(0.00-3.00)		0.375(0.00-3.00)		0.017(0.00-3.00)		1.021(0.00-3.00)	
N3	0.500(0.00-3.00)		0.100(0.00-1.60)		0.135(0.00-2.32)		2.325(0.00-3.00)	
**M-stage**								
M0	0.658(0.00-3.00)	0.633	0.312(0.00-3.00)	0.828	0.570(0.00-3.00)	0.493	1.150(0.00-3.00)	0.609
M1	0.238(0.00-3.00)		0.460(0.00-2.00)		0.020(0.00-2.32)		2.05(0.02-2.85)	
**Differentiation grade**								
High-moderate	0.671(0.00-3.00)	0.986	1.026(0.00-2.85)	0.579	0.756(0.00-3.00)	0.307	0.600(0.00-3.00)	0.480
Low	0.605(0.00-3.00)		0.140(0.00-3.00)		0.122(0.00-3.00)		1.262(0.00-3.00)	
**Resection margin**								
R0	1.032(0.00-3.00)	0.034	1.020(0.00-3.00)	0.249	1.069(0.00-3.00)	0.236	1.237(0.00-3.00)	0.282
R1	0.010((0.00-3.00)		0.000(0.00-2.18		0.000(0.00-3.00)		0.022(0.00-3.00)	
R2	0.690(0.00-2.85)		0.660(0.00-2.85)		0.970(0.00-2.18)		0.125(0.03-1.80)	
**Location**								
Esophagus	0.375(0.00-3.00)	0.094	-		-		-	
GE-junction	0.342(0.00-3.00)		-		-		-	
Stomach	1.150(0.00-3.00)		-		-		-	

### Prognostic significance of PIGR expression

**Figure 3:**
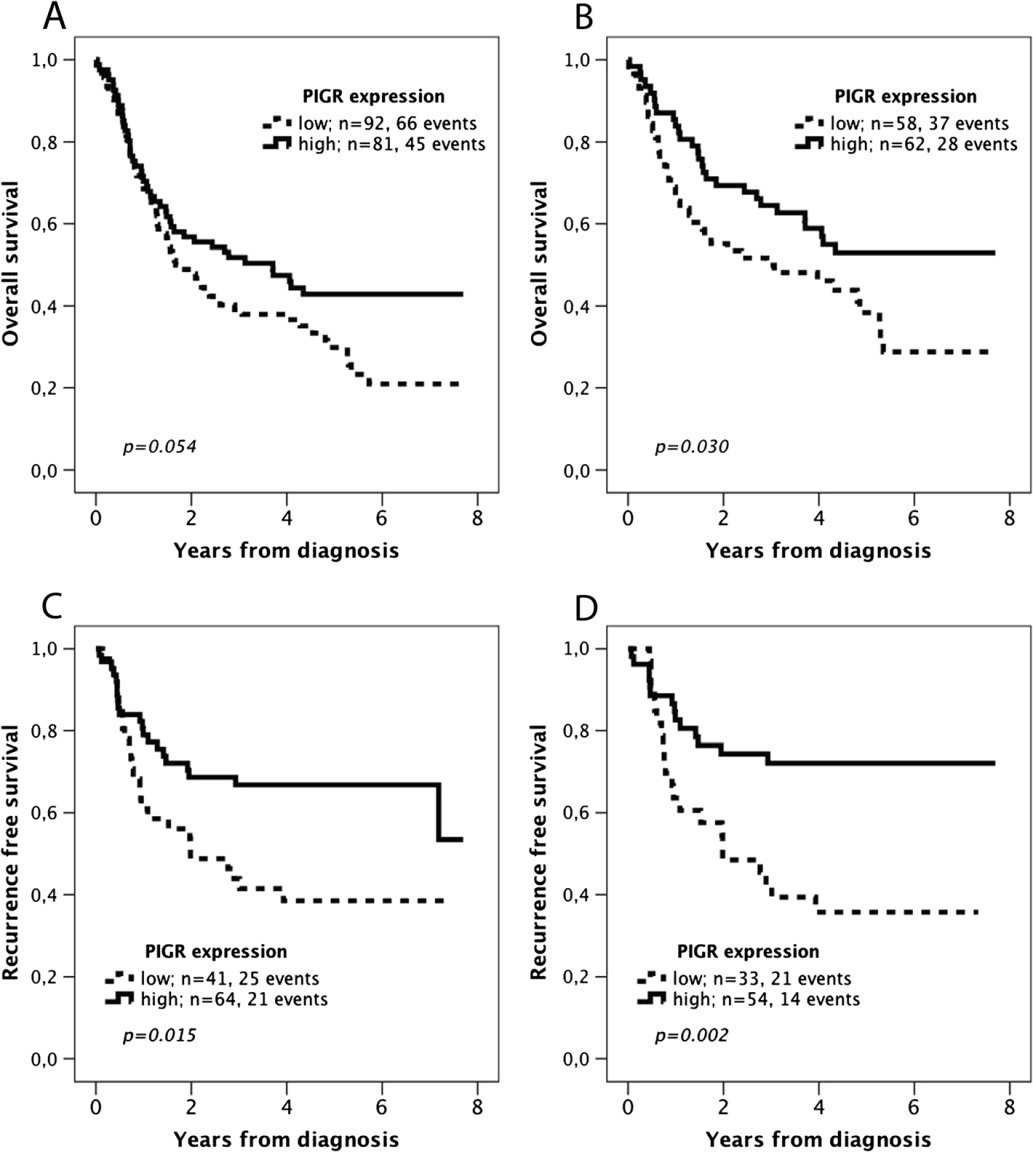
**Kaplan-Meier estimates of overall and recurrence free survival according to PIGR expression.** Overall survival according to PIGR expression in **(A)** the entire cohort, and in **(B)** cases with R0 resection. Recurrence free survival in **(C)** cases with R0 resection, and in **(D)** distant-metastasis free (M0) patients with R0 resection.

According to the results of the CRT analysis a cut off at 0.922 was adopted for OS and 0.356 for RFS (Additional file 1). As demonstrated in Figure 3A, there was a non-significant trend towards an improved OS for cases with high tumour-specific PIGR expression (p = 0.054). In cases with radically resected (R0) primary tumours there was a significant association between high PIGR expression and a prolonged OS (p = 0.030, Figure 3B). There was a significant association between high PIGR expression and an improved RFS in cases with R0 resection (p = 0.015, Figure 3C) and in curatively treated patients with R0 resection and no distant metastases (M0, p = 000.2, Figure 3D). As demonstrated in Table 3, the significant association of PIGR expression and a prolonged OS was confirmed in unadjusted Cox regression analysis (HR 0.58, 95% CI 0.36-0.96, p = 0.032), and remained significant in adjusted analysis (HR 0.60, 95% CI 0.36-0.99, p = 0.044). As further shown in Table 4, PIGR expression was significantly associated with prolonged RFS in unadjusted analysis for cases with R0 resection (HR 0.49, 95% CI 0.27-0.88, p = 0.017) and curatively treated patients with R0 resection/M0 disease (HR 0.37, 95% CI 0.19-0.72, p = 0.004). These associations remained significant in adjusted analysis (HR 0.49, 95% CI 0.27-0.90, p = 0.021 and HR 0.32, 95% CI 0.15-0.69, p = 0.004, respectively).

**Table 3:** Relative risks of death according to clinicopathological factors and PIGR expression – entire cohort and curatively treated patients with radically resected primary tumours

		**Entire cohort**				**R0 resection**				
	**n (events)**	**Unadjusted HR (95% CI)**	**p-value**	**Adjusted HR (95% CI)**	**p-value**	**n (events)**	**Unadjusted HR (95% CI)**	**p-value**	**Adjusted HR (95% CI)**	**p-value**
**Age**										
Continuous	173	1.03 (1.01-1.05)	<0.001	1.04 (1.03-1.06)	<0.001	120 (65)	1.05 (1.02-1.07)	<0.001	1.07 (1.04-1.09)	<0.001
**Gender**										
Female	41 (29)	1.00		1.00		26 (15)	1.00		1.00	
Male	132 (82)	0.75 (0.49-1.14)	0.177	1.16 (0.11-1.90)	0.544	94 (50)	0.85 (0.48-1.52)	0.584	1.05 (0.57-1.94)	0.882
**T-stage**										
T1	18 (5)	1.00		1.00		18 (5)	1.00		1.00	
T2	32 (17)	2.30 (0.85-6.25)	0.102	1.36 (0.47-3.88)	0.568	31 (17)	2.43 (0.89-6.60)	0.082	1.39 (0.46-4.26)	0.560
T3	93 (66)	3.79 (1.52-9.44)	0.004	1.43 (0.51-3.96)	0.495	55 (34)	2.94 (1.15-7.53)	0.025	1.23 (0.40-3.72)	0.716
T4	27 (21)	5.59 (2.09-14.90)	0.001	2.12 (0.72-6.28)	0.175	15 (9)	3.34 (1.11-9.99)	0.031	1.30 (0.34-4.96)	0.697
**N-stage**										
N0	58 (27)	1.00		1.00		46 (18)	1.00		1.00	
N1	29 (17)	1.41 (0.77-2.59)	0.266	1.61 (0.86-2.98)	0.133	22 (11)	1.40 (0.66-2.97)	0.380	1.95 (0.90-4.21)	0.089
N2	41 (30)	2.06 (1.22-3.47)	0.007	2.28 (1.32-3.93)	0.003	27 (17)	2.03 (1.04-3.94)	0.037	3.02 (1.51-6.05)	0.002
N3	45 (37)	3.22 (1.94-5.33)	<0.001	3.14 (1.81-5.45)	<0.001	25 (19)	3.45 (1.80-6.64)	<0.001	4.95 (2.46-9.93)	<0.001
**M-stage**										
M0	136 (83)	1.00		1.00		97 (50)	1.00		1.00	
M1	18 (16)	2.23 (1.30-3.82)	0.004	1.69 (0.95-3.00)	0.072	10 (9)	2.78 (1.36-5.67)	0.005	1.54 (0.67-3.53)	0.311
**Differentiation**										
High-moderate	46 (26)	1.00		1.00		37 (19)	1.00		1.00	
Low	102 (75)	1.50 (0.96-2.34)	0.077	1.36 (0.85-2.17)	0.204	65 (40)	1.23 (0.71-2.12)	0.466	1.42 (0.81-2.49)	0.226
**Margins**										
R0	123 (68)	1.00		1.00			1.00		1.00	
R1	31 (27)	2.75 (1.76-4.30)	<0.001	2.15 (1.34-3.46)	0.002		-		-	
R2	19 (16)	2.34 (1.35-4.07)	0.003	2.31 (1.30-4.11)	0.004		-		-	
**Location**										
Esophagus	59 (33)	1.00		1.00		37 (16)	1.00		1.00	
GE-junction	45 (31)	1.32 (0.81-2.16)	0.266	1.51 (0.90-2.56)	0.120	30 (18)	1.44 (0.74-2.83)	0.286	1.26 (0.62-2.58)	0.523
Stomach	65 (44)	1.26 (0.80-1.94)	0.311	1.59 (0.96-2.63)	0.069	50 (29)	1.44 (0.78-2.66)	0.237	1.41 (0.63-3.16)	0.409
**PIGR expression**										
Low	92 (66)	1.00		1.00		37 (58)	1.00		1.00	
High	81 (45)	0.69 (0.47-1.01)	0.056	1.00 (0.66-1.52)	0.992	28 (62)	0.58 (0.36-0.96)	0.032	0.60 (0.36-0.99)	0.044

**Table 4:** Relative risks of recurrence according to clinicopathological factors and PIGR expression in radically resected primary tumours (R0) and in curatively treated patients (R0 + M0)

		**R0 resection**				**R0 resection + M0**				
	**n (events)**	**Unadjusted HR (95% CI)**	**p-value**	**Adjusted HR (95% CI)**	**p-value**	**n (events)**	**Unadjusted HR (95% CI)**	**p-value**	**Adjusted HR (95% CI)**	**p-value**
**Age**										
Continuous	105 (46)	1.00 (0.98-1.03)	0.728	1.04 (1.01-1.08)	0.005	87 (35)	1.00 (0.97-1.03)	0.887	1.04 (1.00-1.07)	0.049
**Gender**										
Female	20 (5)	1.00		1.00		16 (3)	1.00		1.00	
Male	85 (41)	2.04 (0.81-5.18)	0.132	1.97 (0.73-5.36)	0.183	71 (32)	2.78 (0.85-9.07)	0.091	4.43 (1.21-16.24)	0.025
**T-stage**										
T1	16 (3)	1.00		1.00		11 (1)	1.00		1.00	
T2	28 (10)	2.32 (0.64-8.44)	0.201	1.78 (0.41-7.75)	0.440	24 (7)	3.66 (0.45-29.73)	0.225	2.45 (0.26-22.66)	0.430
T3	48 (25)	3.71 (1.12-12.34)	0.032	1.90 (0.46-7.72)	0.372	43 (22)	7.21 (0.97-53.55)	0.053	3.07 (0.36-26.02)	0.304
T4	12 (8)	6.50 (1.71-24.70)	0.006	2.07 (0.42-10.26)	0.372	8 (5)	11.29 (1.31-97.00)	0.027	4.50 (0.39-51.49)	0.226
**N-stage**										
N0	41 (3)	1.00		1.00		37 (3)	1.00		1.00	
N1	19 (11)	10.52 (2.92-37.85)	<0.001	13.86 (3.72-51.70)	<0.001	17 (10)	9.31 (2.56-33.87)	0.001	10.11 (2.61-39.22)	0.001
N2	26 (16)	12.97 (3.77-44.67)	<0.001	15.68 (4.44-55.41)	<0.001	22 (3)	11.22 (3.19-39.52)	<0.001	14.68 (3.85-55.92)	<0.001
N3	19 (16)	23.32 (6.75-80.56)	<0.001	30.25 (8.48-107.93)	<0.001	11 (9)	19.22 (5.13-71.98)	<0.001	46.878 (10.56-208.038)	<0.001
**M-stage**										
M0	87 (35)	1.00		1.00			-		-	
M1	7 (6)	4.17 (1.74-10.01)	0.001	2.26 (0.77-6.69)	0.139		-		-	
**Differentiation**										
High-moderate	32 (9)	1.00		1.00		31 (9)	1.00		1.00	
Low	55 (31)	2.31 (1.10-4.86)	0.027	2.59 (1.20-5.61)	0.016	39 (20)	1.91 (0.87-4.19)	0.108	1.20 (0.43-3.34)	0.727
**Location**										
Esophagus	32 (12)					26 (8)	1.00			
GE-junction	27 (15)	1.81 (0.84-3.89)	0.128	1.92 (0.80-4.64)	0.144	25 (13)	2.00 (1.83-0.83-4.84)	0.122	3.22 (1.19-8.75)	0.022
Stomach	44 (19)	1.29 (0.63-2.67)	0.484	1.67 (0.66-4.26)	0.282	35 (14)	1.49 (0.63-3.56)	0.367	3.86 (1.35-11.03)	0.011
**PIGR expression**										
Low	41 (25)	1.00		1.00		33 (21)	1.00		1.00	
High	64 (21)	0.49 (0.27-0.88)	0.017	0.49 (0.27-0.90)	0.021	54 (14)	0.37 (0.19-0.72)	0.004	0.32 (0.15-0.69)	0.004

**Figure 4:**
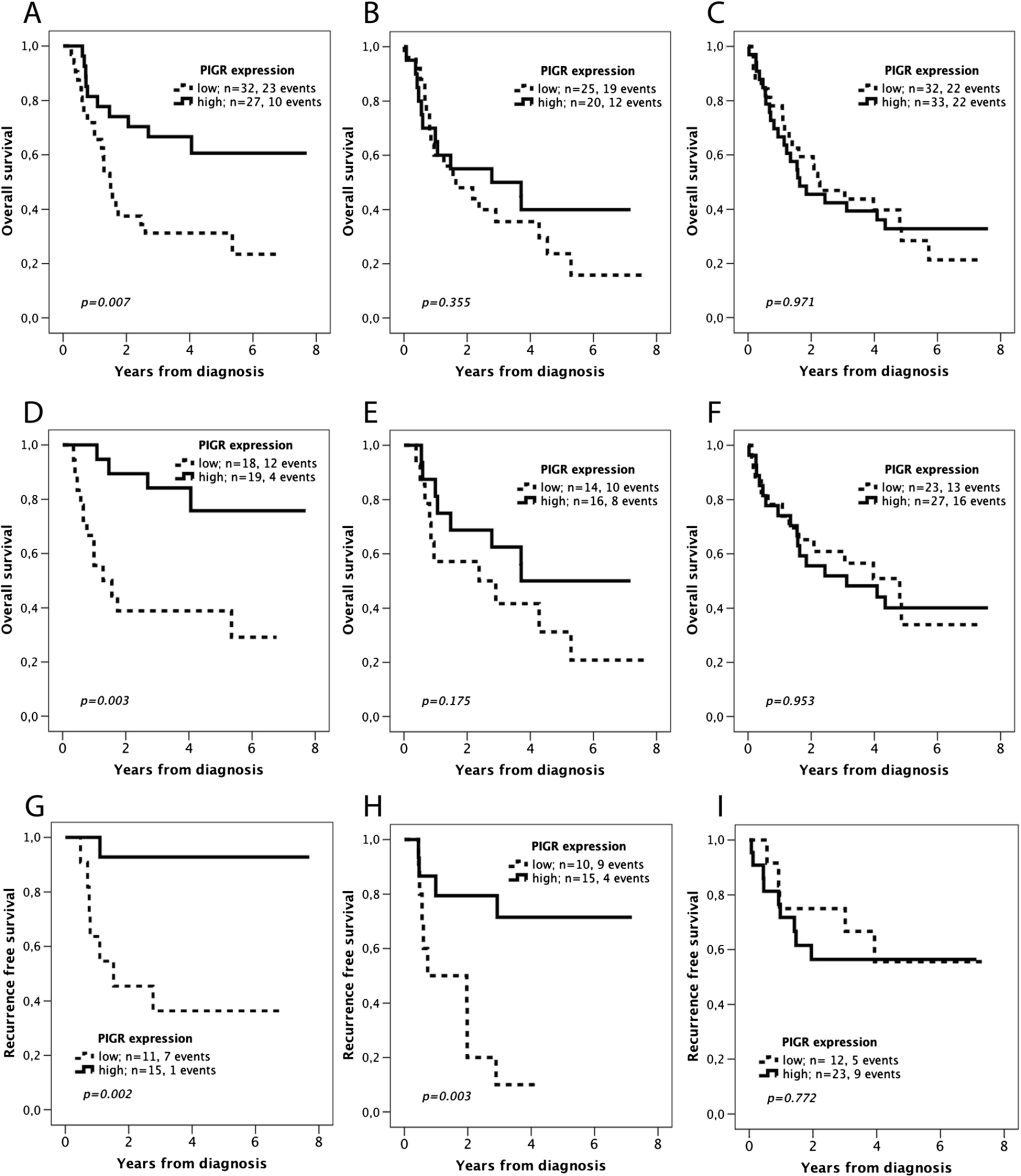
**Kaplan-Meier estimates of overall and recurrence free survival according to PIGR expression in subgroups according to tumour location.** Overall survival in the entire cohort of patients with **(A)** esophageal cancer, **(B)** GE-junction cancer and **(C)** stomach cancer. Overall survival in patients with R0 resection with **(D)** esophageal cancer, **(E)** GE-junction cancer and **(F)** stomach cancer. Recurrence free survival in curatively treated patients/R0 resection with **(G)** esophageal cancer, **(H)** GE-junction cancer and **(I)** stomach cancer.

Subgroup analysis according to anatomical tumour location revealed that the prognostic impact of PIGR expression was most evident in esophageal cancer for OS and esophageal/GE junction cancer for RFS (Figure 4). Of note, tumour location was not prognostic, neither for OS nor RFS (data not shown). PIGR expression did not remain an independent prognostic factor in subgroup analysis according to tumour location (data not shown).

## Discussion

The results from this study demonstrate that high PIGR expression is an independent favourable prognostic factor in adenocarcinoma of the upper gastrointestinal tract. These findings are in line with the study by Gologan et al. encompassing a smaller cohort of 42 adenocarcinomas of the esophagus, GEJ and stomach, where reduced PIGR expression was found to correlate with lymph node metastasis. In the present study, however, there was no significant association between PIGR expression and lymph node metastasis, but high PIGR expression was significantly associated with a less advanced T-stage and uninvolved resection margins. These findings are also in line with the majority of previous studies on other cancer forms, indicating an association between high PIGR expression and a better prognosis; e.g. in colorectal cancer [11], bladder cancer [14], and non-small cell lung cancer [12]. To date, only one study on HCC has demonstrated an association between high PIGR expression and a higher metastatic potential and worse clinical outcome [16].

Comprehensive longitudinal expression analysis revealed that PIGR expression was significantly higher in sampled IM, while PIGR was not expressed in squamous epithelium, and weakly/focally expressed in normal gastric mucosa. These findings are also in line with Gologan et al., where PIGR was found to be uniformly expressed in IM and focally expressed in normal gastric mucosa. Another finding that confirms the results by Gologan et al. is that PIGR expression did not differ in primary tumours/lymph node metastases according to the presence or absence of IM, indicating that PIGR is not associated with carcinogenetic pathways originating in a background of BE or gastric IM. Furthermore, the utility of PIGR as an indicator of high-risk BE or gastric IM is not evident, as the expression did not differ according to the presence/absence of dysplasia, nor by the degree of dysplasia.

There was a tendency towards a lower PIGR expression in lymph node metastases as compared to primary tumours, although these results were not significant. This finding is however in line with the hypothesis that PIGR expression has tumour-inhibiting properties. Furthermore the lack of positive conversion of PIGR expression from the primary tumour to lymph node metastasis, suggests that analysis of PIGR in the primary tumour should be sufficient for prognostication purposes.

Of note, although the independent prognostic value of PIGR expression was retained when adjusting for tumour location in the multivariable model, stratified analysis according to location revealed that the prognostic value of PIGR was largely attributed to tumours located to the esophagus and GEJ, and that PIGR expression was not an independent prognostic factor in separate analysis by tumour location. However, since the number of cases available for analysis in each subgroup was rather small, future studies encompassing tumours from larger patient cohorts are warranted to determine whether the prognostic value of PIGR expression differs by anatomical location in these cancer forms. In this context, the observation of a significant association between PIGR expression and a more advanced N-stage in gastric cancer is noteworthy, since the prognostic value of PIGR was not evident in this category. Nevertheless, it should be acceptable to consider adenocarcinomas of the esophagus, GEJ and stomach as a group in biomarker studies, since their clinical and biological differences and similarities are likely more appropriately distinguished by their molecular characteristics, yet to be better defined, than by their anatomical origin. Moreover, while the distribution of some clinicopathological characteristics differed by anatomical location in the present cohort, adjuvant treatment and survival was similar for all categories.

It should also be pointed out that use of a CRT analysis-derived cut off to determine the prognostic value of PIGR expression may lead to overfitting of the model. Therefore analyses should be regarded as descriptive and the same cut off value should be applied in validatory studies on independent patient cohorts.

In the here analysed retrospective cohort, all cases of neoadjuvant chemotherapy had been excluded and only a minor proportion had received adjuvant chemotherapy. Thus, the favourable prognosis conveyed by a high PIGR expression is not likely due to an adjuvant treatment effect. It would however be of interest to investigate a potential link between PIGR expression and anti-tumoural immune response in future studies and along this line, whether PIGR may predict the response to neoadjuvant or adjuvant chemotherapy [18,19]. Polymorphonuclear neutrophils (PMNs) that are generally believed to be antitumorigenic [20] have also been reported to actually facilitate tumour progression and invasion [21,22]. It is known from previous studies that SC, the extracellular part of PIGR, is able to inhibit interleukin 8 (IL-8) and in turn prevent chemotaxis of PMNs [23]. PMNs activate matrix metalloproteinase-2 (MMP-2), an enzyme involved in angiogenesis [13], tentatively stimulating tumour progression and invasion [21,22]. Thus, an inhibitory effect of MMP-2 by SC could be a possible explanation for the favourable outcome associated with a high tumour-specific PIGR expression.

Since a variety of normal non-B cells and malignant cells have also been found to produce immunoglobulins [24], another interesting avenue of research would be to examine the functional interplay between PIGR and cancer cell-associated immunoglobulins. The accumulated experimental evidence so far indicates that such atypical immunoglobulins promote growth and proliferation of cancer cells [25,26], in turn suggesting that PIGR may regulate these immunoglobulins negatively in the majority of cancer forms, including upper gastrointestinal adenocarcinoma.

A limitation to the present study is the retrospective setting, where curative intent may be difficult to establish. Therefore, we examined the risk of recurrence in relation to PIGR expression in patients having R0 resection and R0 resection/no distant metastases (M0), respectively. In the former category, cases denoted as having metastatic disease had either be operated due to bleeding of the primary tumour (with metastatic disease present) or had non-locoregional lymph node metastases (M1). In the prospective setting, curative treatment intent can be mandatory for inclusion.

Another potential limitation is the use of the TMA technique for all sampled tissue entities. There was, however, no obvious heterogeneity in PIGR expression between duplicate tissue cores, and of note duplicate cores were obtained from different blocks of the primary tumour and different lymph node metastases in cases with more than one metastasis. Moreover, the TMA technique is now an established tool for biomarker studies with equal or even improved ability to identify associations between investigative biomarkers and clinical outcome [27].

## Conclusions

High PIGR expression is associated with a less advanced T-stage and independently predicts a decreased risk of recurrence and an improved survival in patients with adenocarcinoma of the upper gastrointestinal tract. The clinical relevance as well as the functional basis of these observations merit further study.

## Abbreviations

TMA: Tissue microarray; CRT: Classification regression tree; PIGR: Polymeric immunoglobulin receptor; BE: Barrett’s esophagus; IM: Intestinal metaplasia; HCC: Hepatocellular carcinoma; OS: Overall survival; RFS: Recurrence free survival; HR: Hazard ratio; IHC: Immunohistochemistry; GEJ: Gastroesophageal junction; AC: Adenocarcinoma; PMN: Polymorphonuclear neutrophil; SC: Secretory component; IgA: Immunoglobulin A, IL-8, interleukin 8; MMP-2: Matrix metalloproteinase-2.

## Competing interests

The authors declare that they have no competing interests.

## Authors’ contributions

RF evaluated the immunohistochemical stainings, performed the statistical analyses and drafted the manuscript. AG evaluated the immunohistochemical stainings and assisted with the statistical analysis. CH collected and re-examined clinicopathological data and assisted with TMA construction. BN constructed the tissue micro array and performed the IHC stainings. MU contributed with antibody validation. JE assisted with collection of clinical data. KJ conceived of the study, evaluated the immunohistochemistry, and helped draft the manuscript. All authors read and approved the final manuscript.

## Supplementary Material

Additional file 1 **Classification regression tree analysis for selection of prognostic cutoffs.** (A) Overall survival in the entire cohort and (B) recurrence free survival in curatively treated patients with R0 resection. Click here for file
