# Supplementary figures and images for: Expression and prognostic significance of the polymeric immunoglobulin receptor in esophageal and gastric adenocarcinoma
Source: J Transl Med. 2014 Apr 2;12:83. doi: 10.1186/1479-5876-12-83 (PMC4021601; doi:10.1186/1479-5876-12-83)

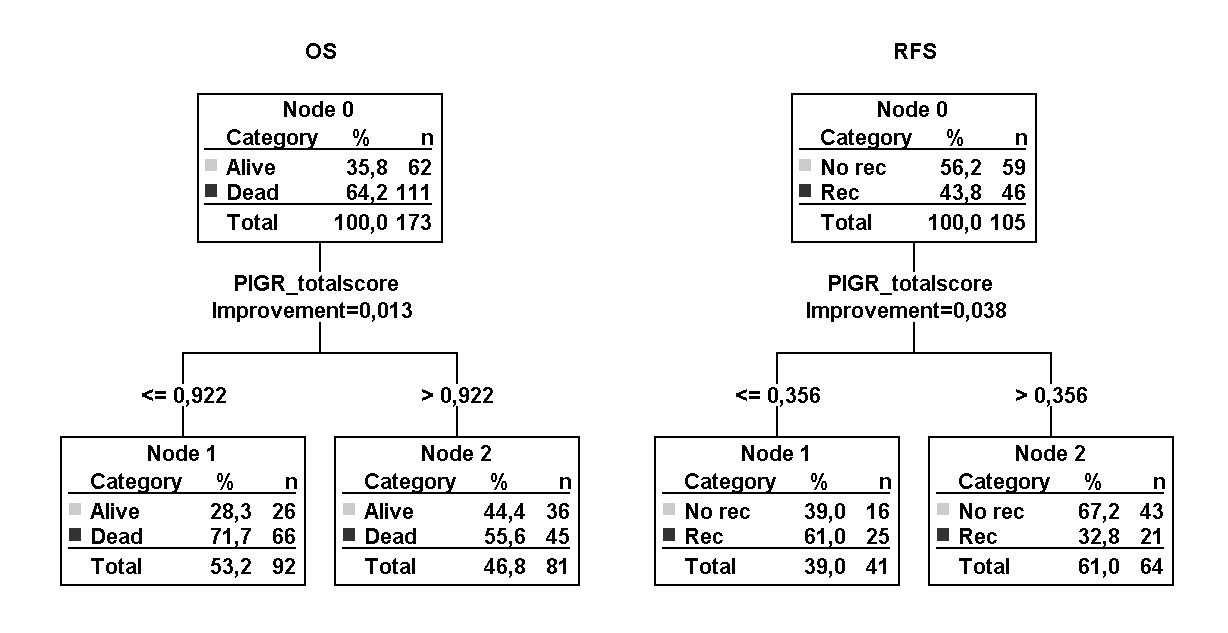

Supplement: Additional file 1 — Classification regression tree analysis for selection of prognostic cutoffs. (A) Overall survival in the entire cohort and (B) recurrence free survival in curatively treated patients with R0 resection. [file 1479-5876-12-83-S1.jpeg]
